# Determining the metabolic fate of human milk oligosaccharides: it may just be more complex than you think?

**DOI:** 10.1017/gmb.2022.8

**Published:** 2022-09-07

**Authors:** Peter Philip James Jackson, Anisha Wijeyesekera, Robert Adrian Rastall

**Affiliations:** Department of Food and Nutritional Sciences, University of Reading, Reading, UK

**Keywords:** Human milk oligosaccharides (HMOs), infants, *Bifidobacterium*, gut microbiota, infant formula, cross-feeding

## Abstract

Human milk oligosaccharides (HMOs) are a class of structurally diverse and complex unconjugated glycans present in breast milk, which act as selective substrates for several genera of select microbes and inhibit the colonisation of pathogenic bacteria. Yet, not all infants are breastfed, instead being fed with formula milks which may or may not contain HMOs. Currently, formula milks only possess two HMOs: 2′-fucosyllactose (2’FL) and lacto-*N*-neotetraose (LNnT), which have been suggested to be similarly effective as human breast milk in supporting age-related growth. However, the *in vivo* evidence regarding their ability to beneficially reduce respiratory infections along with altering the composition of an infant’s microbiota is limited at best. Thus, this review will explore the concept of HMOs and their metabolic fate, and summarise previous *in vitro* and *in vivo* clinical data regarding HMOs, with specific regard to 2’FL and LNnT.

## Introduction: the history of HMOs

Human breast milk is considered the gold standard nutrient source for infants in the early stages of life due to the presence of several remarkable functional ingredients (Van den Abbeele et al., 2019). One group of such ingredients is the human milk oligosaccharides (HMOs). A classification which is given to a group of structurally diverse and complex unconjugated glycans present in human breast milk (Ninonuevo et al., 2006). HMOs were first “discovered” towards the end of the nineteenth century after French biochemist Georges Denigés noted that in addition to lactose, human and bovine milk possessed several other carbohydrate structures with Polonowski and Lespagnol originally terming these unknown fractions as gynolactoses (Polonowski and Lespagnol, 1929, 1931). The levels of HMOs present in breast milk range from 5 to 25 g/L throughout the course of lactation, making the levels of oligosaccharides present in human milk the highest amongst mammalian species. This is over 100 times greater than the oligosaccharide content found in bovine milk, which has been estimated at around 100 mg/L (Robinson, 2019; Zivkovic et al., 2011).

## Structural complexity of HMOs

**Figure 1:**
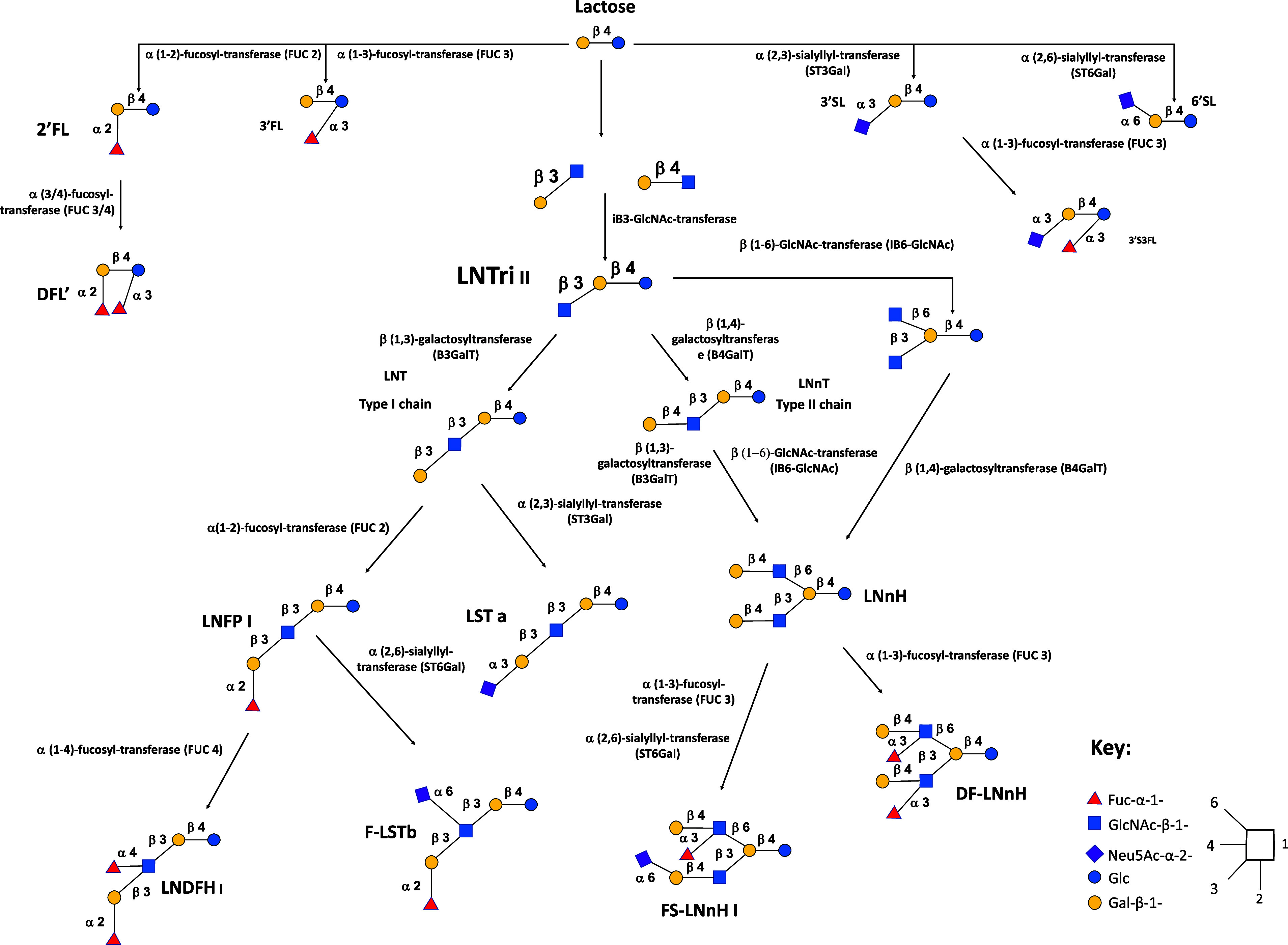
Generalised overview of the complexity and structural diversity of human milk oligosaccharides (HMOs) present in breast milk. Abbreviations: Neutral HMOs: 2’FL, 2′-fucosyllactose; 3’FL, 3′-fucosyllactose; DFL, difucosyllactose; DF-LNnH, difucosylated lacto-*N*-neohexaose; LNDFH I, lacto-*N*-difucohexaitol I; LNFP I, lacto-*N*-fucopentaose I; LNnH, lacto-*N*-neohexaose; LNnT, lacto-*N*-neotetraose; LNT, lacto-*N*-tetraose. *Acidic non-fucosylated HMOs*: 3’SL, 3′-sialyllactose; 6’SL, 6′-sialyllactose; LST a, sialyllacto-*N*-tetraose a. *Acidic fucosylated HMOs*: 3’S3FL, 3’-Sialyl-3-fucosyllactose; F-LST b, sialylfucosyllacto-*N*-tetarose b; FS-LNH I, fucosylsiallacto-*N*-hexose I.

Virtually, all HMOs possess a lactose (Lac) core to which a multitude of different monosaccharide “building blocks,” including galactose (Gal), glucose (Glc), fucose (Fuc), sialic acid (Neu5Ac) and *N*-acetylglucosamine (GlcNAc), can be attached via the action of specific glycosyltransferases in the presence of α-lactalbumin (Smilowitz et al., 2014). The synthesis of HMOs begins with the enzymatic elongation of lactose (Lac) by either β1-3 or β1-6 linkages of Gal to lacto-*N*-biose (LNB) or *N*-acetyllactosamine (LacNAc), respectively. Based on this, HMOs can be classified as either Type-I or Type-II chains. Type-I chain HMOs possess lacto-*N*-tetraose (LNT), which is lactose coupled to LNB. While Type-II chains, are composed LNT isomer lacto-*N*-neotetraose (LNnT), and is Lac linked to LacNAc (James et al., 2016). These core HMO structures can then be further elongated and additionally categorised as neutral, fucosylated or sialylated (Plaza-Diaz et al., 2018; Zivkovic et al., 2011). Neutral HMOs possess structures similar to galacto-oligosaccharides (GOSs) containing both Glc and Gal (Barile and Rastall, 2013), and may also accommodate several GlcNAc or LNB units attached via β1-3 and β1-6 linkages (Ayechu-Muruzabal et al., 2018). At this point, Fuc units can be enzymatically attached via α1-2, α1-3 or α1-4 linkages generating fucosylated HMOs. Hereafter, one or more molecules of Neu5Ac may be attached via α2-3 or α2-6 linkages in the presence of sialyl-transferases generating sialylated HMOs (Smilowitz et al., 2014). Figure 1 gives a generalised overview of the complexity and structural diversity of HMOs present in breast milk.

## Composition of human milk oligosaccharides in breast milk and factors affecting composition

To date, somewhere in the region of 200 HMOs have been identified in the breast milk of mothers with the most widely recognised HMOs being 2′-fucosyllactose (2’FL), LNT and LNnT (Barile and Rastall, 2013; Egge et al., 1983; Urashima et al., 2018). The composition and concentration of HMOs present in breast milk are highly dependent on several critical factors, including geographical location, ethnicity, length of gestation and secretor status. In general, the levels of HMOs present in breast milk are highest immediately following birth and decrease throughout lactation with the concentration of 2′FL appearing to be highest during the first month postpartum (Thurl et al., 2010, 2017; Xu et al., 2017). Yet, the rates at which HMOs decline are not constant across all HMOs. For example, while 2’FL, difucosyllactose (DFL), lacto-*N*-fucopentaose-2 (LNFP II), 3’-Sialyllactose (3’SL) and 6’-Sialyllactose (6′SL) all decline in concentration throughout lactation, the rate of decline does not appear to significantly alter during days 30–120 (Gabrielli et al., 2011; Spevacek et al., 2015; Thurl et al., 2010), whereas lacto-*N*-fucopentaose-1 (LNFP I) appears to decline just 3 days after birth recording a twofold decline by the end of lactation, respectively (Bao et al., 2013; Smilowitz et al., 2013). Other HMOs, including 3′-fucosyllactose (3’FL), appear to increase in concentration throughout lactation by 1.67–1.8-fold (Austin et al., 2019; Gabrielli et al., 2011; Samuel et al., 2019; Smilowitz et al., 2013).

However, a significant percentage of mothers can only synthesise certain HMOs, including 2’FL, dependant on their secretor status. Secretor status is based on the Lewis blood group and depends on the expression of the specific glycosyltransferases, α1-2-fucosyltransferase (FUT2 encoded by the Se gene) and α1-3/4-fucosyltransferase (FUT3 encoded by the Le gene), and can result in marked differences in which HMOs are synthesised (Blank et al., 2012; Hegar et al., 2019). Table 1 summarises these findings.

**Table 1.** Human milk oligosaccharide composition of breast milk based on the genetic background of the mother. *Source*: Vandenplas et al. (2018).

Gene	Lewis gene +	Lewis gene −
Secretor gene +	Se+ Le+	Se+ Le−
	Secretes all HMOs	Able to secrete 2’FL, 3’FL, LNFP I and LNFP III
Secretor gene −	Se− Le+	Se− Le−
	Able to secrete 3’FL, LNFP-II and LNFP III	Able to secrete 3’FL, LNFP III and LNFP V

However, the Lewis antigen system and secretor status can be described as an over generalisation of an extremely complex situation (Plaza-Diaz et al., 2018) as even when these factors are accounted for, substantial differences in HMO profile can still occur. For example, FUT2 and FUT3 have been shown to compete for several of the same substrates. As a result, differences in levels of expression can alter the synthesis of 2’FL by a “secretor” mother (McGuire et al., 2017; Plaza-Diaz et al., 2018).

Additionally, levels and composition of HMOs present in breast milk may also vary between the mothers of preterm and full-term infants regardless of secretor status. However, reports on this are contradictory with several papers seemingly reporting that HMOs may or may not vary between preterm and term infant mothers (Austin et al., 2019; De Leoz et al., 2012; Kunz and Rudloff, 2017; Thurl et al., 2017).

Furthermore, the sizeable heterogeneity in the levels and composition of HMOs detected in breast milk may be in part due to differences in analytical techniques used in studies, including high-performance liquid chromatography mass spectrometry (HPLCMS) and HPLC time-of-flight mass spectrometry, high-performance anion-exchange chromatography with pulsed amperometric detection (HPAEC-PAD), as well as differences in the number of preparation steps taken (van Leeuwen, 2019; Zivkovic et al., 2011). A further complication is the lack of analytical standards needed in order to quantitatively assess the concentration of HMOs present (Wicinski et al., 2020). As a result, it could be argued that determining the average composition and quantity of HMOs in breast milk, based on current published information, is not achievable due to too many confounding variables. Furthermore, to reiterate (Thurl et al., 2017), to be able to determine the average composition of HMOs in breast milk, a worldwide multicentre study following the same protocols would be required. However, even if the average HMO content of breast milk was determined, what relevance this would have regarding clinical significance remains uncertain.

## Human milk oligosaccharides, health benefits and clinical data

It is well documented that breastfeeding is highly associated with several health benefits, including improved growth rate, lower prevalence of respiratory, intestinal and urinary infections (Li et al., 2014) and lower incidence of allergies and autoimmune conditions, with some of this being put down to the presence of HMOs in breast milk (Doherty et al., 2018; Triantis et al., 2018).

The structural nature of HMOs renders them resistant to digestive enzymes found within the GI tract (Garrido et al., 2012a) with >90 per cent of HMOs reaching the colon intact. HMOs likely function as prebiotics, stimulating the growth of beneficial bacteria, including bifidobacteria (Barile and Rastall, 2013; Bode, 2009). HMOs can also act as soluble decoys, preventing the adhesion of pathogenic bacteria to cell surface receptors due to their resemblance to the glycans found on the surface of epithelial cells in the intestinal tract (Wicinski et al., 2020). In addition, the formation of the short-chain fatty acids (SCFAs) butyrate, acetate and propionate from saccharolytic fermentation plays vital roles in the activation and differentiation of immune cells and may also reduce the risk of infections and allergies (Ayechu-Muruzabal et al., 2018; Kumari and Kozyrskyj, 2017). Additionally, it has been shown in infants that a lack of bifidobacteria, particularly those associated with HMO degradation and utilisation, is associated with increases in systemic inflammation and immune dysfunction (Henrick et al., 2021). Furthermore, supplementation of 2’FL in combination with *B. pseudocatenulatum* MP80 was associated with changes in gene expression of both anti-inflammatory and pro-inflammatory markers in the cecum of healthy mice, whereas in mice with dextran sulphate sodium (DSS)-induced colitis, supplementation of 2’FL in combination with *B. pseudocatenulatum* MP80 resulted in attenuations of bodyweight loss, along with a reduction in DSS-induced immune cell infiltration, increase in colon length and disrupted mucosal architecture along with preventing a reduction in occludin expression (Heiss et al., 2021).

However, not all infants are breastfed, with many being fed formula milk which may or may not be supplemented with HMOs. The first two HMOs to be commercially produced were 2’FL and LNnT, and while these compounds are referred to as HMOs, they are not sourced from human milk, but are produced by microbial fermentation using strains of *E. coli* and yeasts which have been genetically modified (Sprenger et al., 2017).

Several studies have suggested that infants fed HMO-supplemented formula milk present lower risks of parent-reported bronchitis and respiratory infections, along with reduced use of antibiotics, less waking at nights and improved age-appropriate growth (Marriage et al., 2015; Puccio et al., 2017). In a study on the 2’FL, solely or in combination with *Bifidobacterium longum* subsp*. infantis* (Bi-26), on cognitive and structural development in young pigs (Sutkus et al., 2022), the authors noted that synbiotic administration of 2’FL and Bi-26 had several interactive effects on microstructural brain components; however, it appeared to have no effect on memory. Preclinical *in vitro* and *in vivo* studies propose that both 2′FL and LNnT promote the growth of several *Bifidobacterium* and *Bacteroides* species, strains, and subspecies including *B. longum* subsp*. infantis, Bacteroides fragilis*, and *Bacteroides vulgatus*, amongst others (Marcobal et al., 2010; Yu et al., 2013).

However, while the importance of breast milk on infant health outcomes is well supported, there remains a great deal unknown regarding the efficacy of HMOs added to infant formulas on infant health outcomes. Furthermore, it has been demonstrated that several species, strains and subspecies of microorganisms found within the gut microbiota, including *Bifidobacterium adolescentis* and *Bifidobacterium animalis*, do not grow well on HMOs, including 2′FL and LNnT on their own (Lawson et al., 2020; LoCascio et al., 2010; Marcobal et al., 2010; Sela et al., 2012; Xiao et al., 2010). This suggests that the composition of gut microbiota may be key if formula milks containing 2’FL and LNnT are to be effectively utilised. Therefore, the rest of this review will focus on the effects that HMOs have on the composition of the infant gut microbiota both *in vitro* and *in vivo* and will attempt to determine the metabolic fate of HMOs.

## The metabolic fate of human milk oligosaccharides: infant and rodent studies

It was established as early as the 1970s that HMOs are present in the urine of expecting mothers as soon as 8 weeks (Hallgren et al., 1977) and can be detected in the mammary glands using ^13^C isotopes (Dotz et al., 2014), suggesting that HMOs circulate throughout maternal serum. Hirschmugl et al. (2019) investigated individual and temporal variations in the composition and levels of HMOs in maternal serum throughout the course of pregnancy. In this study, serum samples were collected from healthy pregnant woman throughout the course of gestation at weeks 10–14 (Visit 1), 20–24 (Visit 2) and 30–35 (Visit 3) and at the time of admission to delivery at the Department of Obstetrics, Medical University of Graz, Graz, Austria. In total, 16 HMOs [2′FL, 3’FL, DF’, 3′SL, 6′SL, LNT, LNnT, LNFP I, II, and III, LST a, b, and c, LNDFH, LNH and disialyllacto-*N*-tetraose (DSLNT)] were detected in serum with the authors reporting a steady increase in the presence of HMOs, in particular, fucosylated HMOs in circulation throughout the course of pregnancy.

Based on this, one could speculate that HMOs may undergo maternal-to-foetal transport. Indeed, there are data suggesting that several HMOs, including 2′FL, 3’FL, DFL and 6′SL, were present in amniotic fluid (Wise et al., 2018). Likewise, it has also been demonstrated, using an *ex vivo* experimental model involving isolated placental cotyledons perfused with 2’FL, that 2’FL was able to cross the placenta (Hirschmugl et al., 2019). Furthermore, given that HMOs function as signalling molecules and that similar glycan structures can act as receptors in cells and tissues (Bhargava et al., 2012), it is likely that HMOs may also contribute towards the development and functioning of immune (Plaza-Diaz et al., 2018) and endothelial cells (Donovan and Comstock, 2016) of infants *in utero.* However, since foetal circulation and cord blood are not accessible during pregnancy, these findings should be interpreted with a great deal of care (Hornef and Penders, 2017; Walker et al., 2017).

To date, the ability of HMOs to end up in the urine of infants has been extensively studied using ^13^C-labelled glycans along with several analytical techniques, including MALDI-TOF-MS, HPAEC-PAD, nano-LC–MS and MALDI FT-ICR MS. The results of these studies suggest that fully and partially intact HMOs, including 2’FL, 3’SL, 6’SL, LNT and LNnT, can be present in the urine of infants, albeit in far lower concentrations than that found in milk at just 4 per cent, with secretor/non-secretor status massively impacting on which HMOs are detected (Borewicz et al., 2020; De Leoz et al., 2013; Dotz et al., 2014, 2015; Goehring et al., 2014).

Given that HMOs can end up in the urine of infants, HMOs can clearly be absorbed through the epithelial cells of the gastrointestinal tract and enter the bloodstream (Dotz et al., 2014; Rudloff et al., 2012). To date, the potential for HMOs to enter circulation has been extensively studied in animal models. In one study, conducted in rats fed both mixed and isolated HMOs, including 2’FL, it was noted that when mixed HMOs were ingested, 2’FL was detected in circulation 30 min later in a dose-dependent manner, reaching a maximum at 60 min (Vazquez et al., 2017), whereas in rats fed isolated HMOs, levels of 2’FL detected in circulation similarly increased in concentration over time, again in a dose-dependent manner. However, they did not reach a maximum peak within the 4-h sampling period. In contrast, Jantscher-Krenn et al. (2013) reported that only 3’SL, along with very few other HMOs, was detected in the serum of rats. The discrepancies in findings between these studies probably result from biological differences in the metabolism of HMOs due to the ages of rats used in each respective study.

The potential for HMOs to enter into the circulation and plasma of infants was investigated by Ruhaak et al. (2014) in 13 full-term infants using solid-phase extraction followed by an analysis by nHPLC-PGC-chip-TOF-MS. In total, 15 oligosaccharides, including LNT, LDFP, LNFT, 3’SL, 6’SL, 3′sialyllactosamine (3’SLN), 6′sialyllactosamine (6’SLN), LNFP III and 2’FL, were detected in the plasma of infants, albeit in lower concentrations than levels found in breast and formula milk, with over a 10-fold variation in LNT being recorded between partially breastfed and formula-fed infants. Interestingly, an unknown isomer of SLN was found. Given SLN is usually derived from bovine milk (McGrath et al., 2016; Ruhaak et al., 2014) and the relative abundances detected in the infants of this study far exceed those found in bovine milk (Fong et al., 2011). From this, one could theorise that new SLNs detected in the circulation of infants may have been produced via interactions between milk, milk by-products and bacterial glycosidases (Lis-Kuberka and Orczyk-Pawilowicz, 2019).

Moreover, using isotopically labelled standards, coupled with ultra-performance liquid chromatography and HPLC, Goehring et al. (2014) investigated the presence of HMOs in both breastfed and formula-fed infants. The authors reported the detection of several HMOs, including 2′FL, 3’FL and LNnT, in the plasma of breastfed but not formula-fed infants, again albeit in far lower levels. A similar finding was reported by Radzanowski et al. (2013), who documented that the concentration of HMOs present in infant plasma was substantially less (3′SL: 0.10–0.78 mg/L; 6′SL: 0.05–0.68 mg/L; 2′FL: 0–2.25 g/L) compared with breast milk (3′SL: 54.3–225 mg/L; 6′SL: 29.3–726 mg/L; 2′FL: 0–3.8 g/L) at just 0.1 per cent, respectively.

## The degradation and transportation of HMOs

**Figure 2:**
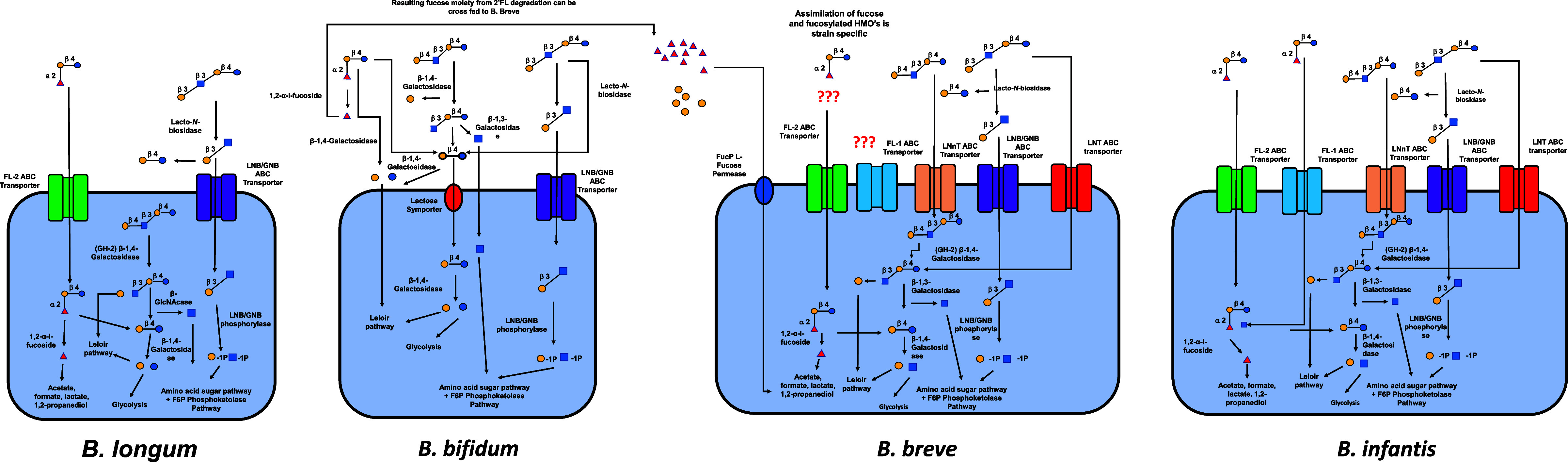
Intracellular and extracellular degradation of the three main human milk oligosaccharides (2’FL, LNT and LNnT) and resulting metabolites by four common species of *Bifidobacterium* (*Bifidobacterium longum, B. bifidium, B. breve and B. longum*) and selective fermentation pathways. Abbreviations: ABC, ATP-binding cassette; FL, fucosyllactose; GNB, galacto-*N*-biose; LNB, lacto-*N*-biose; LNnT, lacto-*N*-neotetraos.

As previously discussed, virtually, all HMOs possess a lactose core to which a multitude of different monosaccharide “building blocks,” including Gal, Glc, Fuc, sialic acid (Neu5Ac) and *N*-acetylglucosamine (GlcNAc), can be attached via the action of specific glycosyltransferases in the presence of α-lactalbumin (Smilowitz et al., 2014). In order to stimulate the fructose 6-phosphate phosphoketolase-dependent glycolytic pathway more commonly termed the bifid shunt active in bifidobacteria, these complex milk glycans must be degraded (Pokusaeva et al., 2011). The mechanisms by which HMOs undergo degradation can be characterised as intracellular (transport-dependant) and extracellular (glycosidase-dependent). Both mechanisms require the use of specific ATP (adenosine triphosphate)-binding cassette (ABC) transporters to either import intact or processed glycans inside the bacterial cell (Garrido et al., 2011) with the most common strains of bifidobacteria (*B. breve, B. longum and Bifidobacterium bifidum*) preferring to utilise specific mechanisms of action (Sakanaka et al., 2020). See Figure 2.

### Fucosidases and fucosylated human milk oligosaccharide transporters

To hydrolyse fucosidic-linked HMOs, two distinct glycoside hydrolase (GH) families, GH95 and GH29, are required for the degradation of specific fucosidic linkages (Sakanaka et al., 2019). While both GH95 and GH29 can target the 1,2-, 1,3- and 1-4-α-l-fucosides, GH29 displays a higher affinity towards both 1,3- and 1,4-α-l-fucosides, whereas GH95 displays a higher preference towards the hydrolysis of 1,2-α-l-fucosides (Matsuki et al., 2016; Shani et al., 2022; Zeuner et al., 2018). The transporters responsible for the uptake of fucosyllactose (FL’) were first discovered in *B. longum* subsp*. infantis* ATCC 15697T (Sela et al., 2008). *B. longum* subsp*. infantis* ATCC 15697T possesses two paralogous FL’ transporters which share up to 60 per cent of the same solute-binding proteins (SBPs), suggesting that there is some degree of overlap in their ability to transport various HMOs (Sakanaka et al., 2020). This overlap was demonstrated in a recent study conducted by Sakanaka et al. (2019) with the authors concluding that FL transporter-1 was only able to import low molecular weight HMOs, including 2′FL and 3’FL. In contrast, FL transporter-2 was able to import not only 2′FL and 3’FL, but also LDFT and LNFP I. Yet, the ability of different species and strains of bifidobacteria to express FL’ transporters is not identical. As research by Garrido et al. (2016) and Matsuki et al. (2016) revealed, there was a remarkable difference in the ability of *B. longum* subsp*. infantis* and *B. bifidum* to utilise FL’ due to the presence of different intracellular and extracellular ABC-type transporters (K02025 and K02026).

Outside of *Bifidobacterium* spp., GH29 and GH95 have also been detected in *Roseburia inulinivorans* and GH29 detected in *Akkermansia muciniphila*, enabling it to cleave the α1-2-fucosyl linkage to Gal (Kostopoulos et al., 2020).

### Lacto-N-biosidase and LNB transporter

LNT is one of the most abundant neutral HMOs present in breast milk and is hydrolysed by lacto-*N*-biosidase (lnb), resulting in the formation of LNB and Lac (Yamada et al., 2017). The specificity of lnb present in *Bifidobacterium* also varies remarkably between species (Sakanaka et al., 2020). On this basis, lnb present in *B. bifidum* is classified as GH20, whereas in *B. longum*, lnb is categorised as GH136. The differences being that GH136 requires an additional chaperonin for proper protein folding and is also capable of accepting sialyllacto-*N*-tetraose a (LST a) in addition to LNT (Sakurama et al., 2013; Yamada et al., 2017).

In *B. bifidum,* degradation of HMOs to LNB and monosaccharides begins extracellularly with the hydrolysis of LNFP I, II and lacto-*N*-difucohexaose I, II (LNDFH I, II) to LNT and Fuc with the aid of additional fucosidases (Marcobal and Sonnenburg, 2012). LNB is then hydrolysed from LNT by Lnb and then transported inside of the cell leaving any remaining fucosyl residues behind. Lnb phosphorylase then converts LNB into its respective monosaccharides Gal and GlcNAc, which then undergo further assimilation (Zivkovic et al., 2011). In *B. longum,* the degradation of LNT seemingly follows similar principles to that of *B. bifidum* with LNB being extracellularly hydrolysed from LNT. However, unlike *B. bifidum*, the hydrolysis of LNB from LNFP I by *B. longum* occurs intracellularly, meaning that no Fuc residues are left behind (Xiao et al., 2010; Yamada et al., 2017). In contrast to both *B. bifidum* and *B. longum,* the degradation of LNT and LNnT to LNB by *B. longum* subsp*. infantis* occurs entirely intracellularly with LNB constituent monosaccharides Gal and GlcNAc entering selective fermentation pathways (Ozcan and Sela, 2018). More recently, the presence of GH136 has been discovered in *Roseburia* with the degradation of fuscoylated pentose and hexose HMOs said to occur extracellularly (Pichler et al., 2020).

### Sialidase

For microorganisms to be able to utilise sialic acids, they must possess the necessary sialidases required to hydrolyse the α-2,3 and α-2,6 linkages of sialylated HMOs (Kiyohara et al., 2011; Zivkovic et al., 2011). In bifidobacteria, the most common sialidases are classified as GH33 with each member of the GH33 family exhibiting preference for specific sialic acid linkages. For example, in *B. bifidum*, the sialidase present is categorised as SiaBb2 and exhibits a preference for α-2,3 linkages, whereas the sialidase present in *B. longum* subsp*. infantis* is classified as NanH2, but unlike SiaBb2 appears to exhibit an equal preference for both α-2,3 and α-2,6 linkages (Juge et al., 2016).

### LNT β-1,3-galactosidase

In bifidobacteria, β-1,3-galactosidases are categorised as GH42 and GH35 and are responsible for cleaving HMOs possessing β-1,3 Gal linkages, displaying hydrolytic ability for both Type-I and Type-II chains with highest activity being displayed on LNT, followed by Lac, LNB and LNnT (James et al., 2016; Yoshida et al., 2012).

### β-1,4-galactosidase

Several other glycosidases for the assimilation of HMOs exist, including β-1,4-galactosidases (Zeuner et al., 2019). In *B. bifidum and B. breve,* the β-1,4-galactosidases belong to the GH2 family and are categorised as BbgIII and LacZ2 and LacZ6, respectively, and are responsible for the hydrolysis of HMOs possessing Lac and Type-II chains (James et al., 2016; Yoshida et al., 2012). This mechanism of HMOs degradation is particularly prominent in *B. bifidum* and functions extracellularly cleaving LNnT at its Galβ-1,4 residue liberating Gal and LNT. Thereafter, LNT is further hydrolysed producing GlcNAc and lactose with lactose undergoing additional hydrolysis resulting in the formation of Glc and Gal, respectively (James et al., 2016; Miwa et al., 2010).

### β-D-hexosaminidases

*N*-acetyl-β-D-hexosaminidases are another set of hydrolytic enzymes, which belong to the GH20 family with three enzymes seemingly responsible for the hydrolysis of HMOs. *N*-acetyl-β-D-hexosaminidases have been detected in *B. longum* subsp*. infantis* (Garrido et al., 2012b) with these GHs being categorised as Blon_2355 and Blon_0732 and Blon_0459. Blon_2355 displays a preference for GlcNAc β1-3 Gal linkages, whereas Blon_0732 and Blon_0459 can additionally release GlcNAc from branched HMOs characterised by GlcNAc β1-6 Gal linkages (Garrido et al., 2012b). Additionally, *N*-acetyl-β-D-hexosaminidases have been discovered in *B. bifidum* JCM 1254, categorised as BbhI and BbhII, with BbhI being shown to hydrolyse lacto-*N*-triose II into GlcNAc and lactose, respectively (Miwa et al., 2010).

It is clear that different species, strains and subspecies of *Bifidobacterium* possess several different mechanisms for the assimilation of HMOs and it is likely that substantial differences in rates of consumption and fermentation occur.

## The *in vitro* assimilation and consumption of HMOs

Differences in the consumption behaviour between different microorganisms found within in the gut microbiota have been assessed *in vitro* using either pooled or singular HMOs as sole carbon sources. To date, bifidobacteria are the most widely studied microorganisms, both singular and in combination, in relation to their ability to utilise HMOs due to their predominance in the breastfed infant gut microbiota. Yet, it is not only bifidobacteria that have the ability to degrade HMOs, several other genera within the gut, including *Bacteroides*, *Roseburia* and *Akkermansia* amongst others, appear to play a role in HMO utilisation.

### Single cultures

As mentioned, bifidobacteria are one of the most well-documented microorganisms in the gut regarding HMO consumption. In one study, conducted by Gotoh et al. (2018), the authors aimed to determine the ability of various strains of bifidobacteria to utilise HMOs, including 2’FL. The four strains of *B. bifidum* tested (JCM1254, JCM7004, TMC3108 and TMC3115) were isolated from either infant faecal samples or obtained from other researchers at the Riken Bioresource centre and subjected to *in vitro* assays using GAM broth and with sugar (HMOs) analysis and concentration being analysed in the spent media. There was a large amount of Fuc and Lac in the supernatants of all four *B. bifidum strains* assimilating both HMOs and Lac by 15 h, with the degradation of 2’FL beginning even before cells entered the exponential phase. This suggests that each strain of *B. bifidum* possessed the necessary 1,2-α-fucosidase and 1-3- and 1-4-α-fucosidases required for the degradation of fucosylated HMOs. Furthermore, the fermentation of 2’FL appeared to occur rapidly and extracellularly which may have important implications regarding the utilisation of HMOs within bifidobacterial communities and subsequent microbial diversity (Sakanaka et al., 2020).

Garrido et al. (2015) also examined the ability of several strains of *B. longum* subsp*. infantis* and *B. bifidum* utilised from breastfed infants to utilise HMOs, including 2’FL, 3’FL, 6’SL and LNT, as sole carbon sources. All *B. longum* subsp*. infantis* strains displayed excellent growth on all HMOs, while the ability of various *B. bifidum* strains was highly variable with several strains showing little growth on pooled HMOs. These findings are similar to those of Gotoh et al. (2018), who recorded that the growth of *B. bifidum* strains (JCM1254, TMC3108 and TMC3115) resulted in higher cell biomass compared with *B. bifidum* strain JCM7004. There is clearly a remarkable variability in the ability of *Bifidobacterium* strains, even of the same species, to utilise HMOs with several species, strains and subspecies appearing to be better adapted than others (Sakanaka et al., 2020).

The differences in the ability of *Bifidobacterium* to utilise HMOs were also demonstrated by Bunesova et al. (2016), who tested several strains of *Bifidobacterium,* including *B. longum* subsp. *infantis, B. longum* subsp *suis* BSM11–5 and *B. bifidum* and *Bifidobacterium kashiwanohense* isolated from the stool samples of 6-month infants. There was substantial variability in the ability of bifidobacterial strains and subspecies to utilise HMOs with *B. longum* subsp. *infantis* being able to utilise 2′-FL, 3′-FL, 3′-SL and LNnT. *B. bifidum* BSM28-1 was able to utilise 6’SL in addition to 2′-FL, 3′-FL, 3′-SL and LNnT, while *B. longum* subsp. *suis* BSM11-5 and *B. kashiwanohense* strains all grew in the presence of 2′FL and 3’FL. Several strains and subspecies of bifidobacteria, however, including *B. bifidum* DSM20215 and *B. breve* DSM 20213, were unable to utilise HMOs to any real degree, with *Bifidobacterium pseudolongum* not being able to utilise HMOs at all. Furthermore, all *B. longum* strains and subspecies tested, including *B. longum* subsp. *suis* BSM11-5 and *B. longum* subsp. *infantis* DSM 20088, were able to utilise the resulting Fuc moiety, albeit with a varying degree of success with *B. kashiwanohense* not being able to metabolise Fuc at all. This adds to the evidence that the composition of the gut microbiota appears to be critical if HMOs are to be effectively utilised.

Ward et al. (2007) also investigated the capability of *Bifidobacterium* to effectively degrade 2’FL and utilise the resulting Fuc and sialic acid moiety with *B. longum bv. infantis* ATCC 15697 achieving both the highest growth rate and the highest Fuc consumption amongst the majority of species and strains of *Bifidobacterium* tested, whereas *B. breve* ATCC 1570 was only able to achieve intermediate levels of growth with only moderate Fuc usage and *B. adolescentis* and *B. bifidum* ATCC 15696 exhibiting no growth on either Fuc or sialic acids. These findings are similar to those recorded by Garrido et al. (2015), who noted that while *B. longum* subsp*. infantis* ATCC 15697 exhibited excellent growth on both 2’FL and 3’FL, *B. bifidum* JCM 7004 only exhibited moderate growth on 2’FL, 3’FL and 6’SL with *B. animalis* JCM 10602 exhibiting no growth, on HMOs at all. Accordingly, Locascio et al. (2007) reported that *B. adolescentis* ATCC 15703 presented little-to-no sign of growth in the presence of several HMOs, whereas Xiao et al. (2010) found that *B. adolescentis* ATCC 15704 and 15705 appeared to be unable to utilise HMOs including LNB. This infers that *B. adolescentis* ATCC 15703, 15704 and 15705 lack the glycosidases and transporters required to assimilate HMOs (LoCascio et al., 2010).

The ability of the *B. longum* subsp. *infantis* ATCC 15697 to effectively utilise HMOs can be put down to the presence of five distinct gene clusters (Sela et al., 2008). In the genomic sequencing of *B. longum* subsp. *infantis* ATCC 15697 a 43-kb gene cluster dedicated to HMO import encoding 21 genes was identified with one of its four loci encompassing all of the necessary sialidases, fucosidases, galactosidases and hexosaminidases required for transporting and metabolising HMOs (LoCascio et al., 2007, 2009; Sela et al., 2008). Several other strains and subspecies of bifidobacteria, including *B. longum* subsp. *longum* DJO10A*B* and *B. adolescentis* ATCC 15703, possess fewer than 11 genes where the lack of SBPs results in an inability to effectively utilise HMOs (Lee et al., 2008; LoCascio et al., 2009).

It is not just *Bifidobacterium* which possesses the ability to utilise and exhibit growth on HMOs. In addition to *Bifidobacterium*, several strains of *Bacteroides*, including *B. fragilis* and *B.* vulgatus, are also known prominent consumers of HMOs (Marcobal et al., 2010). Of these, two strains of *B. fragilis* ATCC2585 can effectively utilise a full range of HMOs, albeit displaying a preference for non-fucosylated HMOs, recording an overall consumption between 25 and 90 per cent. These findings are substantially higher than *B. vulgatus* ATCC8482, which can also utilise a full range of HMOs, again displaying a preference for fucosylated HMOs. This strain displayed much lower consumption rates of total HMOs than *B. fragilis* ATCC2585 at 16–40 per cent, respectively (Marcobal et al., 2010), the difference being the presence of Fuc-specific GH95 and GH29 glycoside hydrolases.

Furthermore, in contrast to *Bifidobacterium* and *Bacteroides,* several strains of Enterobacteriaceae, including EC1000, EC11775, EC29425 and SD13313, appear to be unable to utilise several HMOs, including 2‘FL and 6′SL while displaying limited growth on LNnT. However, these strains could also readily utilise Glc, maltodextrin and GOS as a sole carbon source in pure cultures (Hoeflinger et al., 2015).

### Mixed culture/faecal inoculum

The ability of 2’FL to alter the composition of the gut microbiota has been investigated using an *in vitro* Simulator of Human Intestinal Microbial Ecosystem (SHIME) model using faecal samples from 6-month-old infants had been exclusively formula-fed (Van den Abbeele et al., 2019). The authors noted that 2’FL increased the relative abundance of bifidobacteria *and* butyrate-producing bacteria, shifting the distribution of *Bifidobacterium* spp. from *B. bifidum* towards *B. adolescentis*: an interesting finding given that, as previously discussed, *B. adolescentis* appears to be unable to utilise whole HMOs. This likely indicates that *B. adolescentis* can exploit products of the degradation of HMOs by other microbial community members (Thongaram et al., 2017).

Increases in the concentration of acetate and butyrate were seen in both parts of the distal and proximal colon of the SHIME model, with levels of propionate displaying a more rapid increase in the distal part of the colon upon 2’FL dosing. This is consistent with Vester Boler et al. (2013), who reported that 2’FL was rapidly fermented upon inoculation with mixed faecal cultures. However, the bifidogenic effect of 2’FL appeared to be donor-specific with increases in numbers of bifidobacteria only being observed in the proximal colon of one donor. In another, donor increases in bifidobacteria were observed in both the proximal and distal colon.

Additionally, the authors reported that increases in the concentration of acetate and butyrate detected were seen in both parts of the distal and proximal colon of the SHIME model with levels of propionate recording a more rapid increase in the distal part of the colon upon 2’FL dosing. Thus, from these results, it suggests that the microbiota dependence of individual rates of fermentation of 2’FL is likely to be highly variable between subjects (Marcobal and Sonnenburg, 2012). Yet, the supplementation of 2’FL in this study was undertaken at 2 g/L, approximately twice the concentration of 2‘FL found in formula milks currently for sale on the market (SMA Nutrition, 2020), indicating that the results generated by this study may not give a fair reflection of what might transpire in real life.

In another study conducted by Salli et al. (2019) using faecal samples from healthy infants aged below 1 year, the effects of 2’FL on the composition and metabolites of the infant microbiota were investigated using a semi-continuous colon simulator and was compared against GOSs with Lac as a control. Changes in microbial composition and metabolites were measured via 16S RNA amplicon sequencing and gas chromatography. From the results, it was noted that 2’FL recorded similar increases in numbers of total bacteria, *Firmicutes* and *Actinobacteria* (including bifidobacteria) compared to GOS, but 2’FL was unable to match GOS in reductions of numbers of *Proteobacteria.* Furthermore, levels of SCFAs and lactic acid produced by 2’FL were only half compared with those of GOS, suggesting that at least in this regard GOS results in a greater generation of metabolites associated with beneficial health outcomes than supplementation of 2’FL on its own.

Interestingly, Li et al. (2012) noted that in the *in vitro* fermentation of piglet faeces, LNnT recorded the largest increases in levels of acetate and butyrate compared with FOS, GOS/polydextrose mixture and pooled HMOs, whereas pooled HMOs and FOS recorded higher levels of propionate and lactate. Furthermore, both pooled HMOs and LNnT were able to stimulate changes in the microbial composition, including increasing numbers of total bacteria, *Bifidobacterium, Lactobacillus, B. vulgatus* and *Clostridium* cluster XIVa along with resulting in reductions in *Clostridium* cluster IV, suggesting that both pooled and single HMOs can drive beneficial changes in microbial composition. However, despite differences being detected in microbial composition, both pooled HMOs and LNnT appeared to be no more effective in stimulating changes in microbial composition compared with both FOS and the GOS/polydextrose mixture, respectively.

The metabolic by-products and fermentation characteristics of prebiotics, including GOS, 2’FL, LNnT, 6’SL, high-performance inulin (HP) and gum arabic, were investigated by Vester Boler et al. (2013) using faecal samples isolated from both breast and formula-fed infants using an *in vitro* fermentation model. From the results, it was noted that the rates of fermentation of prebiotics differed significantly between breastfed and formula-fed infants inocula. For example, formula-fed inocula generated higher levels of acetate compared with breastfed inocula (*P* < 0.001) with 6’SL producing the largest concentration of acetate after 12-h fermentation. However, acetate production also varied over time between substrates with GOS generating large quantities of acetate regardless of diet. Butyrate appeared to be less affected by substrate or diet, but was higher in formula-fed inocula compared with breastfed inocula overall (*P* < 0.01); however, no differences were detected at any individual time point. Conversely, propionate was affected by diet, but more so by substrate and time with 6’SL producing large amounts of propionate after 12 h of fermentation. Moreover, the fermentation of 2’FL seemingly levelled off after 6 h, further indicating that 2’FL likely undergoes rapid fermentation upon inoculation (Salli et al., 2019). Finally, regarding microbial composition, numbers of bifidobacteria increased, whereas numbers of *E. coli* and *Clostridium perfringens* decreased regardless of the substrate used.

While using a pH-controlled *in vitro* fermentation model involving faecal donors from healthy, Irritable bowel syndrome (IBS) and ulcerative colitis patients, the most noticeable changes in gut microbiota composition were in *Bifidobacterium* (*P* < 0.01), while supplementation of 2’FL also resulted in increased numbers of *Eubacterium rectale* and *Clostridium coccoides* after 8- and 24-h fermentation [*P* < 0.01 (8 h) and *P* < 0.05 (24 h)] in healthy and [*P* < 0.01 (8 and 24 h)] IBD-ulcerative colitis donors. Significant increases in *Roseburia* at 8 h fermentation were seen in both healthy and IBS but not inflammatory bowel disease (IBD)-ulcerative colitis donors. Interestingly, in both IBD-ulcerative colitis and IBS patients but not healthy donors, there were significant increases in *Atopobium* cluster at 8 and 24 h (*P* < 0.01; Ryan et al., 2021).

These results further add to the evidence that the ability of HMOs and 2’FL to stimulate changes in the microbiota and its resulting metabolites appears to be highly specific and restricted to certain species, strains and subspecies of microbes as well as the initial composition of the gut microbiota (Gotoh et al., 2018; Sakanaka et al., 2020; Yu et al., 2013).

## Cross-feeding: a strategy to ensure dominance?

As previously discussed, the gut microbiota, in particular bifidobacteria, have developed several strategies to colonise and dominate the microbiota of an infant’s gut with some strains, species and subspecies, displaying better potential than others. Interestingly, to help drive the colonisation of the gut, *Bifidobacterium*, *Bacteroides* as well as several other genera, including *Akkermansia*, *Anaerostipes* and *Roseburia*, have developed another strategy based on cross-feeding. Strains and species of *Bifidobacterium* and *Bacteroides* that are not able to utilise whole HMOs can feed on metabolites resulting from exploitation of HMOs by other species and strains (White et al., 2014).

In breastfed infants, *B. bifidum* is said to make up of over 10 per cent of the total number of bifidobacteria present within their gut (Sakanaka et al., 2019). When *B. bifidum* is in abundance, the corresponding microbiota appears to follow suit with higher numbers of several other bifidobacterial species and strains also being recorded (Tannock et al., 2013). The potential for *B. bifidum* to act as cross-feeders for other members of the *Bifidobacterium* genus was noted by Asakuma et al. (2011), who documented that *B. bifidum* left several HMO components, including Fuc and Gal in spent media, indicating that extracellular degradation had occurred and suggesting that non-HMOs utilising species/subspecies may be able to exploit these monosaccharide moieties (Kitaoka, 2012).

Additionally, using faecal suspensions isolated from infants, children and adults in a mucin-based medium supplemented with HMOs, Egan et al. (2014) and Gotoh et al. (2018) recorded the ability of several species and strains of bifidobacteria to grow in the presence and absence of *B. bifidum.* The overall findings of these studies suggest that in faecal suspensions possessing *B. bifidum,* the numbers of several bifidobacteria species and strains, including *B. longum* 105-A and *B. breve* UCC2003, increased; strains not known to effectively utilise whole HMOs to any real extent. It seems that *B. bifidum* is likely to be a prominent player in the establishment of the microbiota in early life (Kitaoka, 2012).

It was reported in a single ecosystem that 2′FL derived metabolites from *B. pseudocatenulatum* strains LH9, LH11, LH13 and LH14 supported the growth of several non-HMOs utilising strains including *B. longum* LH12. However, *B. longum* subsp*. infantis* LH23 2′FL degradation products did not support the growth of *B. breve* (LH21 and LH24), respectively. Additionally, with *B. longum* LH206 2′FL conditioned media, increases in numbers of all strains of *B. longum* subsp*. infantis* and *B. pseudocatenulatum* tested were seen. This indicates that the metabolism of 2′FL by *B. infantis* LH206 may generate a wider variety of growth-promoting compounds (Lawson et al., 2020).

It has been documented in a co-culture experiment that *Anaerostipes cacae* was able to utilise monosaccharides, as well as lactate and acetate, resulting from HMOs degradation by *B. Infantis* (Chia et al., 2021). *Roseburia* spp. were able to grow in the presence of *A. muciniphila*, whereas in pure culture *Roseburia* spp. showed little-to-no sign of growth (Pichler et al., 2020). The ability of *Bacteroides* to act as primary degraders of HMOs was demonstrated in mice-fed sialylated HMOs, including 3’SL and 6’SL, when a marked increase in Enterobacteriaceae was seen. This led to an exacerbation of the pro-inflammatory response (Huang et al., 2015).

Additionally, in antibiotic-treated germ-free mice infected with either *Salmonella typhimurium or C. difficile*, it has been demonstrated that *S. typhimurium* was able to access both Fuc and sialic acid and *C. difficile* was able to readily utilise sialic acid as a result of breakdown of host carbohydrates by *Bacteroides thetaiotaomicron* (Ng et al., 2013). However, while the expansion of enteric bacterial pathogens via the utilisation of HMOs is sometimes seen *in vitro* in co-cultures or using *in vivo* mechanistic disease state rodent models, in the highly complex ecosystem in the human gut, this has never been reported.

Not all microorganisms found within the gut can participate in cross-feeding due to intracellular metabolism of polysaccharides/glycans. The inability of specific bifidobacteria to act as cross-feeders was demonstrated by Garrido et al. (2016). *B. longum* SC596 exhibited excellent growth on both Type-I and Type-II chain HMOs, albeit displaying a preference for fucosylated HMOs; however, no monosaccharides from HMOs degradation were detected in the medium. As the degradation of HMOs by *B. longum* SC596 appears to be similar to that of *B. longum* subsp*. infantis* ATCC 15697, which uses intracellular metabolism (Garrido et al., 2013), this suggests that *B. longum* SC596 cannot partake in the cross-feeding of other microorganisms.

These results suggest that the mutualistic behaviour which exists between microorganisms found in the gut likely influences the rates at which metabolites such as SCFAs are generated (Comstock, 2009). From this, one could conclude that the degree to which this mutualistic behaviour exists between microorganisms found in the gut not only increases the diversity of the gut microbiota, but is maybe one of the most critical characteristics in helping to shape a flexible, healthy ecosystem (Gotoh et al., 2018).

## The influence of human milk oligosaccharides on infant microbiota composition *in vivo*

As previously discussed, the ability of HMOs to alter microbial composition in infants has been studied extensively using *in vitro* test conditions, but less so *in vivo* with only a limited number of studies being undertaken to date.

In a proof-of-concept study (De Leoz et al., 2015), serial faecal samples were collected from two vaginally born infants. Infant A was breastfed directly from birth, whereas infant B received formula supplementation for 4 days from days 2–6 and then was solely breastfed thereafter. Faecal samples were collected twice per week for the first month, twice per month in the second month and once or twice per month thereafter. Microbial compositions were analysed via 16S rRNA sequencing. The results demonstrated that after an initial rise in non-HMO-consuming bacteria, including *Enterobacteriaceae* and *Staphylococcaeae,* large shifts in microbial composition from non-HMO-consuming bacteria to HMO-consuming bacteria *Bacteroidaceae* and *Bifidobacteriaceae* were seen. Yet, large differences were seen between both donors whereby week 13 *Bifidobacterium* spp. dominated in infant A, and levels of most faecal HMOs dropped dramatically, whereas by week 14, *Bacteroides* spp. were most dominant in infant B.

Borewicz et al. (2019, 2020) aimed to correlate the HMOs in breast milk with changes in faecal microbiota composition, analysed via Illumina HiSeq 16S rRNA gene sequencing, in healthy 2-, 4-, 6- and 12-week-old breastfed infants. Unsurprisingly, the ability of infants to utilise HMOs, including 2’FL, was associated with differences in the faecal microbiota composition, with those infants possessing relatively high abundances of *Bifidobacterium* 418, 614 and 681 and *Lactobacillus* 744 (FDR < 0.05) reporting higher rates of 2’FL consumption. Additionally, infants who recorded higher consumptions rates of LNT and LNnT, LNFP III, LNFP II and lacto-*N-*hexaose (LNH) possessed significantly higher relative abundances of *Bifidobacterium* OTUs 418, 406, 643, 658, 423, 1335 and 597 and *Bacteroides* 144 (*FDR* < 0.05). Moreover, the degradation of sialylated HMOs 3′SL, 6′SL, LST a, LST b and LST c appeared to be more highly associated with *Bacteroides;* a finding confirming those reported by Yu et al. (2013), who demonstrated using *in vitro* models that *B. fragilis, B. vulgatus* and *B. thetaiotaomicron* could utilise 3’SL and 6’SL as sole carbon sources.

Interestingly*, Borewicz et al. (**2019*, *2020**)* also recorded that lactobacilli appeared to thrive in the presence of 2′FL, DFL, LNDFH I, LNT, LNnT and LNFP II. This is fascinating given that it has been shown repeatedly in several *in vitro* studies that lactobacilli appear to be unable to utilise HMOs (Schwab and Ganzle, 2011; Ward et al., 2006). This suggests that lactobacilli might be able to thrive in the infant’s gut via cross-feeding, scavenging any resulting metabolites, including Fuc and lactose from the extracellular degradation of HMOs (Zuniga et al., 2018). This likely infers that the degradation of HMOs strongly correlates with the microbiota and specifically with the relative abundance of the phylotypes *Bifidobacterium, Bacteroides* and *Lactobacillus* present within an infant’s gut.

Moreover, in a randomised, double-blind, multicentre clinical trial, healthy full-term infants (aged 0–14 days) were fed infant formula with no added HMOs (control), or the same formula with the addition of 2’FL and LNnT for a timeframe of 6 months. Thereafter, all infants were fed the same non-HMO-containing infant formula (Berger et al., 2020). Results were analysed against a breastfed reference group with changes in microbial community types being analysed at 3 and 12 months via 16S rRNA gene sequencing. The results indicated that, compared with the breastfed reference group, the HMO-containing formula stimulated increases in *Bifidobacterium*, albeit to a lower degree than the reference breastfed group. Levels of *Escherichia* were, however, significantly lower in the HMO-containing formula group compared with the control group and were similar to those in the breastfed group. Numbers of *Peptostreptococcaceae* were far higher in the control and HMO-containing formula group compared with the breastfed group. Yet, at 12 months, no differences were detected between the two formula groups. This suggests that the supplementation of infants with HMO-containing formulae may offset some of the ill effects associated with not breastfeeding from birth. However, this study is not without limitation. First, only two faecal samples were collected: one at 3 months and one at 12 months. Second, no data were collected on day-care attendance and when solid foods were introduced (weaning) which, due to the effects these factors have on the microbial composition (McBurney et al., 2019), may have introduced biases into the results. Consequently, further investigation into this area would be highly beneficial to determine the true effects that both 2’FL and LNnT have on altering the composition of healthy infants *in vivo.*

In another study, differences in gut microbiota composition between caesarean and vaginally born babies of α1-2 fucosylated secreting mothers were conducted by Tonon et al. (2021). In this study, faecal microbiota composition from caesarean and vaginally born infants was analysed by 16S rRNA gene sequencing and qPCR with results being stratified by secretor status. The authors concluded that levels of *Bifidobacterium* were similar between caesarean and vaginally born infants of secretor mothers. Yet, there were differences between caesarean and vaginally born infant microbiotas with the caesarean born infants from secretors possessing higher amounts of *Kluyvera* and *Veillonella.* Vaginally born infants from secretor mothers possessed higher amounts of *Bacteroides.* This further adds to the evidence that mode of delivery may likely impact on proliferation of the gut microbiota and HMO utilisation.

In addition to healthy infant’s, the effects of HMOs on the gut microbiota and health outcomes also been studied in preterm infants. In one study, 12 premature infants were randomised into two groups – one group containing formula and increasing doses of short-chain GOSs (degree of polymerisation < 8) and the other group receiving formula + HMOs (Underwood et al., 2014). The authors noted that relative abundances of clostridia increased with increasing doses of HMOs. The authors also noted that there were trends towards increase of γ-proteobacteria over time/dose in preterm infants feed formula + HMOs.

Additionally, in a study involving preterm infants with necrotising enterocolitis (NEC), it was noted that infants with NEC possessed higher levels of *Proteobacteria* and lower levels of *Actinobacteria* at phylum levels, along with lower relative abundances of *B. longum* and higher relative abundances of *Enterobacter cloacae.* The authors also noted that the composition of breast milk, specifically lower concentration of DSLNT, was associated with the likelihood of developing NEC (Masi et al., 2021). While, similarly, Underwood et al. (2015) concluded that preterm infants of non-secretor mothers possessed higher levels of *Proteobacteria* and lower levels of *Firmicutes*, secretor mothers possess specific fucosylated HMOs including LDFT and LNFP V, which may be associated with lower levels of *Enterobacteriaceae* and potentially protective effect against pathogens associated with NEC. These results potentially infer that the composition of HMOs present in breast milk may be a contributing factor towards the development of NEC in preterm infants.

Moreover, in another study conducted in healthy rats, Chleilat et al. (2020) investigated the effects that the supplementation of 2′FL and 3′SL either together or on their own had on microbial composition compared to a non-HMOs control. In general, all HMO-fortified diets altered gut microbiota composition. However, larger increases in *Bifidobacterium* spp. were recorded in the 2′FL group compared with the 3′SL-fortified group (*P* = 0.03). Additionally, *A. muciniphila* numbers were significantly lower in 3′SL + 2′FL group compared with the control (*P* < 0.01), respectively.

In a study involving mice supplemented with the 2’FL and 2’FL consuming strain *B. pseudocatenulatum* MP80, it was noted that 2’FL created an environment that allowed *B. pseudocatenulatum* MP80 to thrive, along with finding that 2’FL increased the *Bifidobacteriaceae* relative abundance, as well as resulting in higher log ratios of *Bacteroidaceae* and *Bifidobacteriaceae* relative to *Lachnospiraceae* and *Ruminococcaceae* amplicon sequencing variant (*P* = 0.003).

These results help to explain the large variability in the presence and levels of HMOs detected in the faecal samples of infants, even when secretor status is considered, with virtually no HMOs being detected in faecal samples of several infants, whereas, in the faecal samples of other infants, there was a strong presence of non-fucosylated HMOs, suggesting the presence of the fucoside-utilising microorganisms needed to degrade fucosylated HMOs (Asakuma et al., 2011). In faecal samples of several other infants, large quantities of LNnT were detected with LNnT not being detected in others. However, despite these differences, a common characteristic amongst nearly all infants used in these studies was what appeared to be the presence of several new, non or partially intact HMOs and HMOs by-products in faecal samples with the majority of new HMOs detected displaying a high proportion of HexNAc (Davis et al., 2016; De Leoz et al., 2013; Dotz et al., 2015).

Thus, while results seemingly imply that the supplementation of 2’FL and LNnT may contribute towards a positive shift in the composition of the gut microbiota, in reality, the make-up of the infant’s microbiota is shaped through several often complex and interacting factors from birth, including mode of delivery (vaginal vs. c-section), feeding practices (breast vs. bottle feeding) and age at which the introduction of complex dietary substrates occurs (weaning). The use of gut microbiome altering medications and supplements, namely antibiotics and probiotics (Bertelsen et al., 2016; McBurney et al., 2019), will also have an impact. Consequently, the degradation of HMOs will differ greatly depending on the relative abundance of specific species, strains and subspecies of microorganisms present within an individual infant’s gut microbiome. More detailed analysis is needed for infant microbiota prior to supplementation in such studies. The supplementation of 2’FL and LNnT in infant formula milk may be of little-to-no benefit to many infants as several infants especially those who were never breastfed may not possess the necessary microorganisms and thus glycosidases and transporters needed to effectively utilise these specific HMOs.

## Research transparency and reproducibility

The opinions/views contained within this article are those of the authors.
